# Feasibility and Reliability of the AWIN Welfare Assessment Protocol for Dairy Goats in Semi-extensive Farming Conditions

**DOI:** 10.3389/fvets.2021.731927

**Published:** 2021-10-21

**Authors:** Monica Battini, Manuela Renna, Mauro Giammarino, Luca Battaglini, Silvana Mattiello

**Affiliations:** ^1^Department of Agricultural and Environmental Sciences—Production, Landscape, Agroenergy, University of Milan, Milan, Italy; ^2^Department of Veterinary Sciences, University of Turin, Turin, Italy; ^3^Department of Prevention, ASL TO3, Veterinary Service, Turin, Italy; ^4^Department of Agricultural, Forest and Food Sciences, University of Turin, Turin, Italy

**Keywords:** animal welfare indicators, *Capra hircus*, extensive husbandry systems, feasibility, inter-observer reliability

## Abstract

The aim of this study was to test the feasibility and reliability of the Animal Welfare Indicators (AWIN) protocol for welfare assessment of dairy goats when applied to semi-extensive farming conditions. We recruited 13 farms located in the NW Italian Alps where three assessors individually and independently applied a modified version of the AWIN welfare assessment protocol for goats integrated with some indicators derived from the AWIN welfare assessment protocol for sheep. The applied protocol consisted of nine individual-level (body condition score, hair coat condition, abscesses, overgrown claws, udder asymmetry, fecal soiling, nasal discharge, ocular discharge, and improper disbudding) and seven group-level (severe lameness, Qualitative Behavior Assessment-QBA, thermal stress, oblivion, Familiar Human Approach Test-FHAT, synchrony at grazing, synchrony at resting) animal-based indicators. On most farms, the level of welfare was good. Many of the considered welfare problems (overgrown claws, fecal soiling, discharges, and thermal stress) were never recorded. However, oblivion, severe lameness, hair coat condition and abscesses were detected on some farms, with percentages ranging from 5 to 35%. The mean percentage of animals with normal body condition was 67.9 ± 5.7. The level of synchronization during resting was on average low (14.3 ± 7.2%). The application of the whole protocol required more than 4 h/farm and 3 min/goat. The inter-observer reliability varied from excellent (udder asymmetry, overgrown claws, discharges, synchrony at resting, use of shelter) to acceptable (abscesses, fecal soiling, and oblivion), but insufficient for hair coat condition, improper disbudding, synchrony at grazing, QBA. Differences in background of the assessors and feasibility constraints (i.e., use of binoculars in unfenced pastures, individual-level assessment conducted during the morning milking in narrow and dark pens, difficulties when using the scan and instantaneous sampling method due to the high number of animals that moved at the same time) can affect the reliability of data collection. Extensive training seems necessary for properly scoring animals when applying the QBA, whereas the FHAT to evaluate the Human-Animal Relationship of goats at pasture seems promising but needs to be validated. Indicators that evaluate the synchrony of activities require to be validated to identify the best moment to perform the observations during the day.

## Introduction

Welfare assessment in extensive production systems has received a lower interest in research than in intensive husbandry systems (1). This is partly due to the belief that animals in open ranges live a more natural life, hence welfare issues are perceived as a minor risk (2). It is undeniable that farm animals at pasture can express a fuller behavioral repertoire, exercise during the day and enjoy the benefit of sun (3). However, the efforts to adapt to outdoor conditions can be costly, for example in terms of thermoregulatory activity and fulfillment of nutritional requirements. Therefore, extensive livestock systems require specific indicators in order to properly assess the welfare of animals, considering the variety of issues they may face (1). Furthermore, differently from intensive husbandry systems that are quite similar across Europe and industrialized countries, pasture-based systems present an extreme variability in relation to the environmental context, and therefore they require a wider range of indicators that encompass all the possible contexts of application (4).

There are at least three issues that need to be considered in case of welfare assessment in extensive conditions: (1) although valid indicators for welfare assessment in intensive/indoor husbandry systems are already available, many of them still need to be tested for validity under extensive conditions and, in some cases, new indicators need to be identified (5–7); (2) the feasibility of data collection may be compromised due to different management and environmental conditions in outdoor systems (e.g., adverse climatic conditions or difficulty to restrain animals for close examination); (3) data collection under these difficult conditions may affect the reliability of the results (e.g., the assessor may not be able to reach the animals for close inspection, and might be forced to use optical instruments for inspecting animals at distance). Therefore, in spite of the fact that feasibility traits of indicators and their reliability are fundamental pre-requisites that determine the effective application of a protocol (8), these last two issues present possible constraints when assessing animal welfare in open ranges.

Compared to other species [e.g. cattle, pigs; (9)], goats are more often raised in developing countries or marginal areas (Asia—especially China, India, Bangladesh, Pakistan, Africa, and Middle East), mainly in smallholder and mixed farming systems. Worldwide, goats are an important component of pastoralist herds. Even in Europe, goat farming is common in marginal areas, where other agricultural activities would be impracticable, and therefore is an important activity which limits the abandonment of such areas (10).

Even if there is a need for assessing the welfare of goats in extensive systems, the scarcity of the research on this topic (1) makes it difficult to find suitable indicators to develop a valid and comprehensive welfare assessment protocol. A recent review on welfare assessment of ruminants at pasture identified 33 animal-based indicators for cattle and 20 for small ruminants, namely sheep and goats (11). However, only three of these indicators were developed and tested specifically for goats in extensive conditions: Qualitative Behavior Assessment (12), Body Condition Score (13), and body weight (13).

In 2011–2015, an EU-funded project on Animal Welfare Indicators (AWIN) developed on-farm welfare assessment protocols for sheep, goats, horses, donkeys and turkeys, possibly using animal-based indicators (14, 15). Despite a common approach, each AWIN protocol has its own characteristics and target category depending on the species. To give an example, the sheep protocol is intended for adult ewes, both for milk and meat production, bred indoor and/or outdoor, whereas the goat protocol was only developed for adult dairy goats in intensive (defined as those in which goats are permanently kept indoors and diet is mainly composed of preserved forages and concentrate) or semi-intensive (similar to the intensive ones, but with occasional access to pasture) husbandry systems (16, 17). These systems differ from those that rely almost exclusively on natural resources for feeding, with no or limited access to housing structures (extensive systems) or from those that rely mainly on pasture, with limited use of feed supplements in periods of greatest need, and the presence of facilities for sheltering animals in case of need (semi-extensive systems).

Research on the assessment of animal welfare in goats kept in semi-extensive and extensive systems is relatively new. An attempt to compare the application of the AWIN protocol in semi-intensive and extensive husbandry systems was carried out in Brazil on meat goat does (18). Since the AWIN welfare assessment protocol was developed for intensive dairy goat farms, in that study the authors only retained few indicators from the original protocol, added some from the AWIN welfare assessment protocol for sheep, and some indicators were partly modified and/or proposed *ex novo*. Unfortunately, the research performed by Leite Oliveira et al. (18) does not clarify the process that led to the selection and/or exclusion of some indicators from the goat protocol, nor if the indicators extrapolated from the sheep protocol were reliable also for meat goats. However, this study provides useful information about the feasibility of such protocol under extensive farming conditions in Brazil. An adapted version of the AWIN welfare assessment protocol was applied to 41 farms housing double-purpose goats in Central Portugal (19, 20). The farms included in that study reflected the husbandry systems of Portuguese rural areas: goats were housed at night in sheds or stables underneath farmers' houses and taken to pasture almost every day, in mixed flocks together with sheep. The authors stated that the removal of few animal-based indicators (e.g., queueing at feeding and at drinking) and the addition of few resource- and management-based indicators to the original AWIN protocol increased the suitability of this protocol to the context, making it more feasible. The concurrent validity of some newly introduced indicators, such as the number of days at pasture, was verified based on its relationship with already validated animal-based indicators, such as the prevalence of overgrown claws. Although the reliability of the protocol was not specifically evaluated, the new indicators are supposed to be reliable, as they consist mainly of easy-to-collect resource and management information.

The aim of this research is to test the feasibility and reliability of a protocol for welfare assessment of dairy goats in semi-extensive systems, which are commonly found in the Italian Alps, using a modified version of the AWIN welfare assessment protocol for goats, integrated with some indicators derived from the sheep protocol.

## Materials and Methods

### Farms

Goat farms were extracted from a database of 163 farms, provided by the ASL (Local Health Center) TO3 territory of Pinerolo-Collegno (Province of Turin, Piedmont, NW Italy). From the database, we selected farms presenting the following characteristics: (i) raising goats for dairy or dual purposes; (ii) making use of outdoor grazing in spring and autumn in proximity to the winter housing buildings; (iii) keeping goats only, with no coexistence with other domestic species; (iv) breeding prevalently Alpine and Valdostana breeds (and their crossbreeds, Alpine × Valdostana); (v) voluntary acceptance of the farmer. Only 13 farms satisfied all the inclusion criteria and were therefore included in the survey. None of the selected farms bred animals for dual purpose; hence, the assessment was only performed on dairy animals.

These farms housed the goats during the winter in indoor pens or, on three farms, in tie stalls. During spring and autumn, the animals were housed during the night and they were released in flat to medium/steep slope areas (ranging from 470 to 920 m a.s.l.) near the farms after the morning milking, giving them the opportunity to graze in meadows, but also to browse the surrounding bushy and woody areas. In some farms, only bushes and woods were available for foraging. In nine farms, woods were also used as shelters to protect the goats from wind, sun, and rain. When woods were not available, the goats had no shelters. On average the total area available for spring and autumn pasture was equal to 20,872.73 m^2^, but large differences were found among the farms (min: 110 m^2^; max: >100,000 m^2^). The average available pasture area/goat was equal to 343.47 m^2^ (SD: 502.82 m^2^; min: 3.55 m^2^; max: 1,470.60 m^2^). The goats had access to pasture for 90–250 days/year for 4–12 h/day, except in one farm where they had permanent access to the outdoor grazing area. During summer, the goats were taken to alpine ranges from June to October for a total period of 90–180 days.

The total number of goats in our farm sample ranged from 12 to 77 with a mean (±SD) of 31.2 (±20.74) goats. Lactating goats ranged from 8 to 77 animals, with a mean (±SD) of 17.70 (±18.71) goats. The average age of lactating goats ranged from 24 to 78 months. Goats were in their mid-lactation stage and were milked twice a day. In 12 farms, the goats were manually milked, while one farm was provided with a mobile milking unit. Besides making use of fresh grass and bushes available in the grazing area, once or twice a day, during milking, eight farms provided supplementary feed consisting of hay, chestnuts, alfalfa, bran, whole or flaked barley, and whole or flaked corn. Three farms provided supplementary feed consisting of whole, flaked or mash corn, with or without mineral supplementation, in the winter period only. In two farms, no supplementary feed was delivered. Fresh and clean water was always available inside the barn. At pasture, water was available through streams (five farms) or watering tanks (four farms); water was not available at pasture in four farms. The distance of water from the pasture area ranged from 0 to 1,500 m.

Claw trimming occurred once a year in six farms, when necessary in five farms, every 6 months in one farm and every 4 months in another farm.

All the farms produced cheese from pure goat milk in small dairies adjacent to the farms. The cheeses were sold at the farm shop, at local markets or were destined to the small-scale organized distribution.

### Assessors

In each farm, the welfare assessment protocol was tested in the period April–July 2019, during the spring grazing period. In order to test protocol reliability, the assessment was carried out by three assessors who had different background and level of experience with dairy goats. The three assessors were students of the MSc in Animal Science at the University of Turin (Italy). Assessor A also had a M.Sc. in Veterinary Science and in Biostatistics, worked as a veterinarian in the Public Health Service and had more than 10 years of experience with dairy goats. Assessors B and C had no specific experience with dairy goats. The three assessors received a common training before the beginning of the study, including both theoretical and practical sessions, and received the AWIN protocol (17) as training material. The training was given by two authors of the AWIN welfare assessment protocol for goats kept in intensive or semi-intensive production systems.

### Data Collection

Farmers were contacted by telephone to illustrate the research and gather essential information about the farm routine, in order to identify the best time for welfare assessment, which depended mainly on milking time.

On each farm, the protocol was applied simultaneously and individually by the three assessors, without any kind of interaction among them. All the assessors were unknown to the farms. The assessments took place mostly under sunny weather (77%), with some cloudy days but never when raining. Visibility was always good. Wind was almost absent. Ambient temperatures ranged from 9 to 24°C, with an average of 18°C. Relative humidity ranged between 21 and 90%, with an average of 54%.

**Figure 1:**
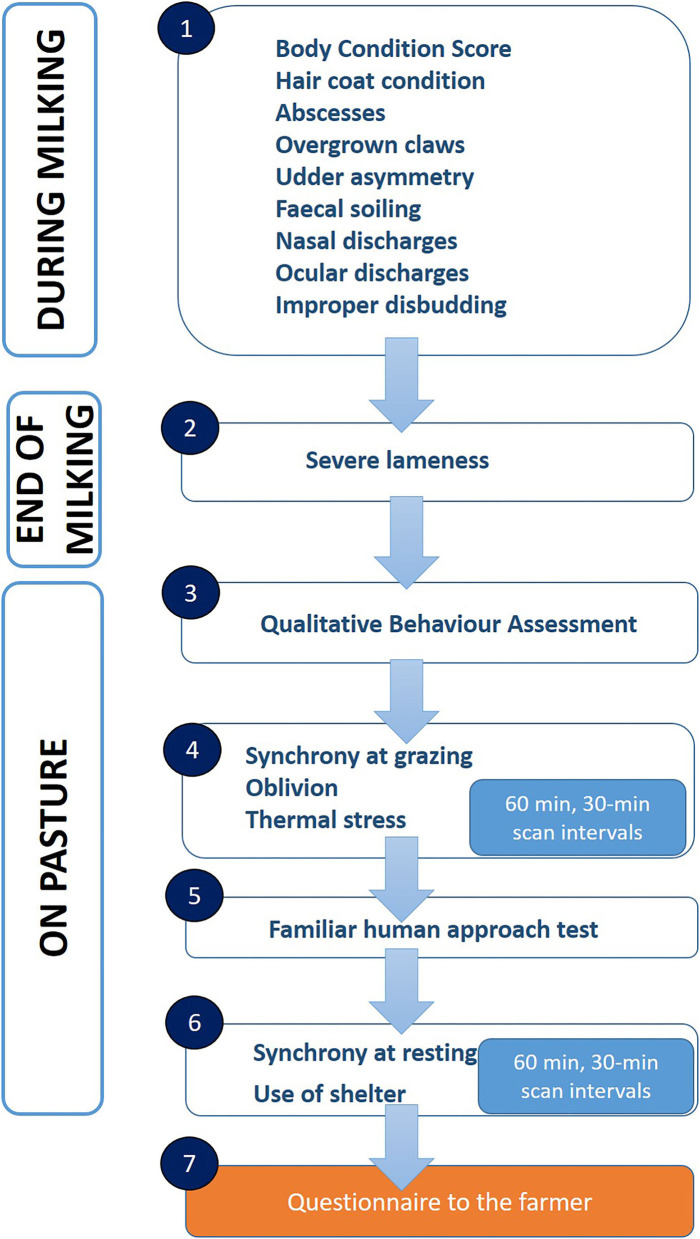
Flowchart of data collection.

The protocol included 13 out of the 18 original indicators from the 2nd level AWIN welfare assessment protocol for goats (17), plus four new indicators. Some of the original indicators had to be adapted to the semi-extensive conditions (Table 1). The whole protocol applied in our study consisted of nine individual-level and seven group-level indicators. Some indicators of the AWIN protocol for goats kept in intensive or semi-intensive production systems were discarded, as they had no meaning or could not be applied in the context of extensive farming (Table 1). The main change from the original AWIN welfare assessment protocol for goats was the order of collection of the indicators that was modified to better adapt to the semi-extensive system, where animals are released outdoors in the morning after the milking routine (Figure 1). The animals were always milked in small pens inside the barn. Data collection started with individual-level assessment during the morning milking of the following indicators: body condition score, hair coat condition, abscesses, overgrown claws, udder asymmetry, fecal soiling, nasal discharge, ocular discharge, and improper disbudding. For these indicators, the same scoring—as detailed in the AWIN protocol for goats—was used (17). At the end of milking, the goats were brought outdoors by the farmer and allowed to graze pasture areas located near the farm. On the way to pasture (walking a distance of 300 m up to 1 km), severe lameness was recorded, based on the observation of abnormal gait, head nodding, spine curvature and kneeling (17). Due to management reasons, lactating goats were mixed with dry goats and yearlings at pasture; hence, the following group-level indicators were recorded both on lactating and non-lactating animals. The assessors first conducted the Qualitative Behavior Assessment (QBA), using the 13 descriptors detailed in the AWIN welfare assessment protocol for goats (17). QBA observations were always performed from one observation point for 10 min on the whole group of goats (12, 23). Then, using a scan sampling method (60 min observation period with 30 min scan intervals, i.e., three scans at time 0, 30, and 60 min), the assessors recorded the number of goats that grazed simultaneously, the number of goats showing signs of thermal stress (shivering or panting), and the number of goats physically or mentally isolated from the group (oblivion) (17). Then, the quality of the human-animal relationship (HAR) was assessed by using a simplified Familiar Human Approach Test (FHAT), following the procedure described in the AWIN welfare assessment protocol for sheep, but in which only the reaction of goats toward the farmer was assessed (avoidance, contact, approach), whereas the distance expressed in meters was not assessed (16). This decision was made because this was a first attempt to apply a test, which has not been validated for goats yet. Assuming that approximately one and a half hour after milking goats start resting, possibly seeking for adequate shelters (24), a second scan session (60 min observation period with 30 min scan intervals) was then used to record the number of goats resting and the number of goats resting in a sheltered place (if present), specifying the type of shelter.

**Table 1:** Indicators applied for refining the AWIN welfare assessment protocol for goats to semi-extensive farming conditions.

**Indicator**	**Description**	**Origin^a^**	**Level^b^**	**Location^c^**	**Notes**
Hair coat condition^1^	Goats with poor hair coat condition (described as: matted, rough, scurfy, uneven, shaggy hair coat, frequently longer than normal) are recorded	A	I	M	Converted to an individual-level indicator (formerly group-level indicator in the AWIN welfare assessment protocol for goats)
Body condition score^2^	BCS is visually assessed at the rear of individual goat, using a three-level scoring method	A	I	M	Retained as it is
Abscesses^1^	The presence of abscesses (ruptured or not) is recorded	A	I	M	Retained as it is from the 2nd level
Overgrown claws^1^	The presence of overgrown rear claws (exceeding the normal length and/or width leading to a loss of the common triangular profile) is visually assessed on individual goats	A	I	M	Retained as it is
Udder asymmetry ^1^	The presence of one half of the udder that is at least 25% longer than the other is recorded	A	I	M	Retained as it is
Fecal soiling^1^	The presence of soft fecal matter below the tail head and on both sides of the tail is visually assessed on individual goats, as a sign of diarrhea	A	I	M	Retained as it is
Nasal discharge^1^	The presence of any mucous or purulent discharge (white or yellowish) from the nose is visually assessed on individual goats	A	I	M	Retained as it is
Ocular discharge^1^	The presence of clearly visible flow from one or two eyes is visually assessed on individual goats	A	I	M	Retained as it is
Improper disbudding^1^	Goats showing presence of residual horns (scurs) are recorded	A	I	M	Converted to an individual-level indicator (formerly group-level indicator in the AWIN welfare assessment protocol for goats)
Severe lameness^1^	Goats showing signs of severe lameness (based on abnormal gait, head nodding, spine curvature, kneeling) are recorded	A	G	T	Assessed when goats were brought to pasture
Qualitative Behavior Assessment (QBA)	The assessor integrates perceived details of behavior, posture and context into the summarization of an animal's style of behaving, or “body language”, using a fixed list of descriptors. List of descriptors: aggressive, agitated, alert, bored, content, curious, fearful, frustrated, irritated, lively, relaxed, sociable, suffering	A	G	P	Retained as it is, but animals can be observed from only one observation point, with sessions lasting maximum 10 min
Synchrony at grazing	The number of goats grazing simultaneously is recorded, using an instantaneous and scan sampling method (60 min observation session, 30 min scan intervals)	N	G	P	Synchronization during grazing is usually evaluated using scan sampling method (21)
Thermal stress	The number of animals showing signs of heat or cold stress is recorded	A	G	P	The indicator was retained as it is, but it was collected using a scan sampling method (60 min observation session, 30 min scan intervals)
Oblivion	The number of oblivious goats is recorded. An oblivious goat is defined as an animal, which is physically or mentally isolated from the group	A	G	P	The indicator was retained as it is, but it was collected using a scan sampling method (60 min observation session, 30 min scan intervals)
Familiar human approach	The closest possible distance of approach from the farmer before an elicited flight response is recorded If no flight response is triggered (goats remain motionless at human approach) this is recorded as 0 m If the goats actively move toward (goats walk directly toward the stockperson) and interact (sniffing, nosing) with the stockperson, this is also recorded	N	G	P	(16)
Synchrony at resting	The number of goats resting simultaneously is recorded, using an instantaneous and scan sampling method (60 min observation session, 30 min scan intervals)	N	G	P	Synchronization during resting is usually evaluated using scan sampling method (22)
Use of shelter	The number of goats resting simultaneously using a shelter is recorded, with instantaneous and scan sampling method (60 min observation session, 30 min scan intervals)	N	G	P	We considered the inclusion of this indicator not only as presence/absence of shelters [as in the AWIN welfare assessment protocol for sheep; (16)], but evaluating the effective use of shelters when present
Queuing at feeding	The number of goats queuing at the feed rack is counted during feeding time, using a scan sampling method during 15 min/observation (2 min/scan)	E			No feed delivered at the feeding rack during spring, but goats have access to pasture
Queuing at drinking	The number of goats queuing at the drinker is counted during feeding time, using a scan sampling method during 15 min/observation (2 min/scan)	E			No drinkers available
Kneeling at the feeding rack	The number of kneeling goats (front legs flexed, the rear up) is counted while they are at the feeding rack	E			No feed delivered at the feeding rack during spring
Latency to first contact test	The time elapsed from when the assessor stops in a pre-determined starting place in the pen and the contact with the first goat that nuzzles or touches any part of the assessor's body is recorded (max time: 300 s). After assessing the Latency to first contact test, the assessor leaves the pen before reentering to perform the Avoidance distance test	E			This test is not applicable outdoors
Bedding	Evaluation of the quantity and cleanliness of the bedding in the pen	E			This test is not applicable outdoors

*When indicators are “Retained as it is” this is referred to the original AWIN welfare assessment protocol for goats. Further specifications are listed in the table*.

1 *Dichotomous categorical variable (absence = 0; presence = 1)*.

2 *Trichotomous categorical variable (very lean = −1; normal body condition = 0; very fat = 1)*.

a *A, indicator retained from (17); N, new indicator, not originally present in (17); E, indicator from (17) which was excluded in the current protocol*.

b *I, individual level; G, group level*.

c *M, during the morning milking; T, during transfer from milking area to pasture area; P, at pasture*.

Assessor A evaluated all the 13 selected farms. Due to unforeseen circumstances, assessors B and C only assessed 12 farms. A minimum of two assessors per farm was always guaranteed.

### Data Analysis

For individual-level indicators, absolute and percentage frequencies of animals without welfare problems were calculated. The prevalence of group-level indicators was calculated as the proportion of goats with absence of welfare problems out of the number of assessed goats. For indicators collected with the scan sampling method, we considered the proportion of goats in the scan with the highest number of animals synchronized during grazing and resting or presenting thermal stress or oblivion, out of the total number of goats. FHAT results were reported as the proportion of farms where goats avoided, accepted the contact or spontaneously approached the farmers out of the total number of farms. Principal Component Analysis (PCA, correlation matrix, no rotation) was used to explore results from the QBA. Data was analyzed using the statistic software IBM SPSS v. 26.0 for Windows (25).

The above-mentioned welfare outcomes are presented in the results referring to data collected by Assessor A. This choice is due not only to the fact that this was the only assessor who evaluated all the farms, but also because this assessor had more experience with goats than the others. Therefore, data collected by Assessor A were used as reference and compared with data collected by the other two observers in order to assess inter-observer reliability (IOR).

IOR was only calculated for the 11 farms where all the three assessors were present. For categorical data (individual-level indicators) IOR was calculated over all animals, regardless of farm, using the S index (26, 27). This index, selected in order to overcome the problem of the paradoxical behavior of Cohen's Kappa, considers the null hypothesis for which the agreement between pairs of observers can be considered as the result due to chance and can be calculated using the following equation:


$$\begin{array}{l} S=\frac{p_{o}-\left(\frac{1}{M}\right)}{1-\left(\frac{1}{M}\right)} \end{array}$$

where *p*_*o*_ is the rate of observed concordance and *M* is the number of categories.

The percentage of concordance agreement was calculated for pairs of assessors against Assessor A (A vs. B and A vs. C) and for the three assessors together (A vs. B vs. C). The S index was only calculated for the three assessors together. For continuous data (group-level indicators), intra-class correlation (ICCs) coefficients were calculated (95% confidence intervals, based on absolute agreement, random effects type, mean-rating). According to Bateson and Martin (28), we adopted the following thresholds to evaluate the quality of reliability: <0.50 = poor; 0.50–0.75 = moderate; 0.76–0.90 = good; >0.90 = excellent.

For QBA, the IOR of Principal Component (PC) scores attributed by the three observers to each farm on the first two PCs was analyzed by using the Kendall Correlation Coefficient *W*. The results were interpreted according to Martin and Bateson (29, 30), where *W*: 0.0–0.2 = slight correlation; 0.2–0.4 = low correlation; 0.4–0.7 = moderate correlation; 0.7–0.9 = high correlation; 0.9–1.0 = very high correlation.

According to several authors (7, 29), a guideline for an acceptable threshold of correlation coefficients when assessing IOR might be set at ≥0.7. Even if the literature report different limits (28–30), our results will be discussed following this guideline.

At the end of the assessment, the assessors were asked to report the major constraints experienced during the application of the protocol.

## Results

The results of the application by Assessor A of the welfare assessment protocol for dairy goats in semi-extensive conditions are shown in Table 2, except for improper disbudding, severe lameness, and FHAT. The proportion of goats properly disbudded is not reported in Table 2, as this procedure was performed in one farm only (73.3% of goats properly disbudded). Severe lameness is not reported as only one goat showing this welfare problem was observed. As to the assessment of HAR quality, FHAT shows that in 61.5% of the farms the goats actively moved toward the farmers and interacted with them (sniffing, nosing). In one farm, the goats remained motionless at human approach, whereas in the remaining 30.8% of the farms the approaching farmer elicited a flight response.

**Table 2:** Absence of welfare problems (mean ± SE; min–max) observed in 13 semi-extensive dairy goat farms, recorded during individual- and group-level assessment.

**Individual-level assessment**		**Group-level assessment**	
**Indicator**	**Mean% ± SE%** **(min%–max%)**	**Indicator**	**Mean% ± SE%** **(min%–max%)**
Normal body condition	67.9 ± 5.69 (25.0–100.0)	Absence of severe lameness	99.4 ± 0.50 (92.3–100.0)
Good hair coat	97.3 ± 1.99 (75.3–100.0)	Synchrony at grazing	92.5 ± 3.63 (60.5–100.0)
Absence of abscesses	88.1 ± 3.09 (65.0–100.0)	Thermal comfort	100.0 ± 0.00 (100.0–100.0)
Regular claws	100.0 ± 0.00 (100.0–100.0)	Absence of oblivious goats	99.3 ± 0.45 (94.9–100.0)
Symmetric udder	96.2 ± 1.44 (87.0–100.0)	Synchrony at resting	14.3 ± 7.22 (0.0–80.0)
Absence of fecal soiling	100.0 ± 0.00 (100.0–100.0)	Use of shelter (out of goats at resting)	95.1 ± 4.86 (56.3–100.0)
Absence of nasal discharge	100.0 ± 0.00 (100.0–100.0)		
Absence of ocular discharge	100.0 ± 0.00 (100.0–100.0)		

**Figure 2:**
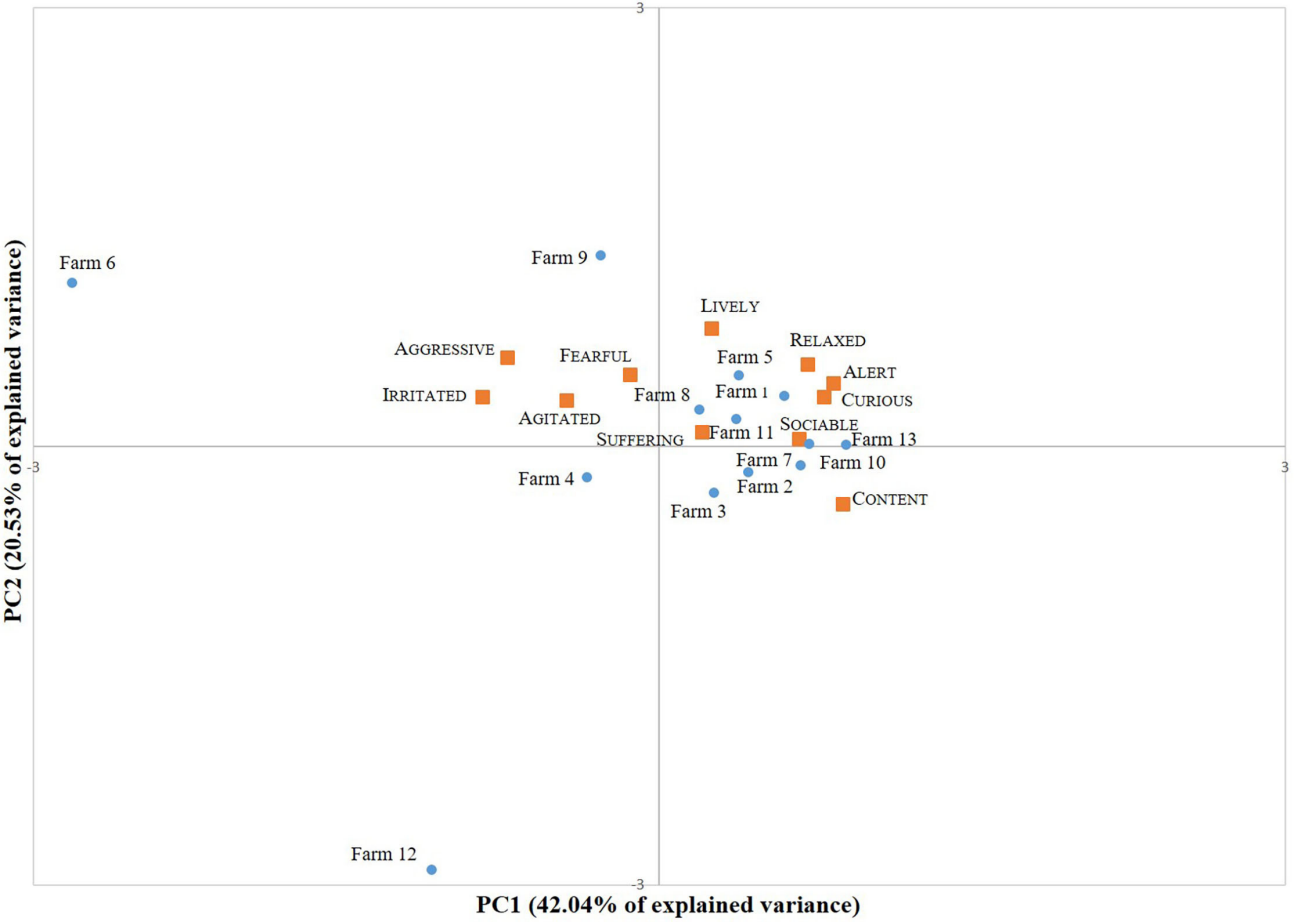
Biplot showing the loadings of the descriptors (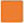) and the farm scores (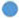) on PC1 and PC2.

Two descriptors (Bored and Frustrated) were scored 0 in all the farms by Assessor A; hence, they were removed from the analysis. PCA was performed on 11 out of 13 descriptors. The analysis identified four main PCs with eigenvalues >1 (4.624, 2.258, 1.753, and 1.080 for PC1, PC2, PC3, and PC4, respectively). The first two PCs together explained 62.57% of the total variance among the farms (PC1: 42.04%; PC2: 20.53%). Figure 2 shows the distribution of the descriptor loadings and of the farm scores along the first two PCs. Descriptors on PC1 (that commonly describes the valence of emotions) range from Content to Irritated, whereas descriptors on PC2 (that describes the arousal) range from Lively to Content.

The IOR calculated for individual-level observations is reported in Table 3. The reliability among assessors appears to be excellent for udder asymmetry, overgrown claws and nasal and ocular discharges, acceptable for abscesses and fecal soiling, but insufficient for hair coat condition and improper disbudding. For group-level indicators results are reported in Table 4 and show that the IOR among assessors is excellent for synchrony at resting and use of shelter, whereas it is acceptable for oblivion (even if the lower limit of confidence interval only reached 0.32) and insufficient for synchrony at grazing.

**Table 3:** Inter-observer reliability for individual-level observations.

**Indicator**	**Concordance agreement (A-B)**		**Concordance agreement (A-C)**		**Concordance agreement (A-B-C)**		**Inter-Observer Reliability (A-B-C)**
	***N***	**%**	***N***	**%**	***N***	**%**	***S* index (LCL-UCL)**
**Body condition score**	153	78.4	214	70.1	137	75.2	S** = 0.81 (0.77–0.85)
Hair coat condition	153	85.0	214	74.8	137	83.0	S* = 0.66 (0.57–0.76)
**Abscesses**	152	94.7	197	83.2	129	90.7	S* = 0.85 (0.80–0.90)
**Overgrown claws**	153	100	214	99.5	137	99.5	S* = 1
**Udder asymmetry**	152	96.1	214	89.3	136	95.6	S* = 0.91 (0.85–0.96)
**Fecal soiling**	152	86.8	213	88.7	135	92.1	S* = 0.85 (0.78–0.92)
**Nasal discharge**	152	100	214	100	136	100	S* = 1
**Ocular discharge**	152	100	214	100	136	100	S* = 1
Improper disbudding	14	71.4	15	80.0	14	76.2	S* = 0.56 (0.20–0.91)

N = sample size; % = percentage of agreement, LCL, Lower Confidence Level; UCL, Upper Confidence Level. S^*^ = S weighted with linear weights; S^**^ = S weighted with square weights, Thresholds: <0.50 = poor; 0.50–0.75 = moderate; 0.76–0.90 = good; >0.90 = excellent. In bold, indicators where correlation coefficient is ≥0.7.

**Table 4:** Inter-observer reliability for group-level observations.

**Indicator**	**Reliability ICC (95% CI)**
Synchrony at grazing	0.64 (−0.44 < CI <0.93)
**Oblivion**	0.74 (0.32 < CI <0.92)
Thermal stress	N.D.
**Synchrony at resting**	0.94 (0.84 < CI <0.98)
**Use of shelter**	1 (0.99 < CI <1)

*ICC, intraclass correlation coefficient; CI, confidence intervals; N.D., not determined*.

*Thresholds: <0.50 = poor; 0.50–0.75 = moderate; 0.76–0.90 = good; >0.90 = excellent. In bold, indicators where correlation coefficient is ≥0.7*.

The agreement among assessors for QBA was considered unacceptable for PC1, due to the rather low Kendall's *W* (0.597). The agreement on PC2 was acceptable (Kendall's *W*: PC2 = 0.750).

The collection of individual-level indicators took about 3 min/goat and the time required to perform the whole protocol was about 4 h/farm. The assessors reported the presence of some constraints that reduced the feasibility of both individual- and group-level indicators. For individual-level indicators, narrow pens and low-light conditions affected the quality of the assessment and extended the time needed for the inspection of the animals; for group-level indicators, open ranges, the presence of thick vegetation cover and the presence of guardian dogs were the major limitations.

## Discussion

This paper reports the results of the application of the AWIN welfare assessment protocol for goats in semi-extensive husbandry systems. The original protocol was partly modified to be adapted to the different context.

The assessment showed that in our farms some welfare problems were completely absent (overgrown claws, fecal soiling, discharges, and thermal stress) or almost absent (severe lameness, synchrony at grazing, and oblivion). However, a comment is necessary for hair coat condition and abscesses. The average situation for both these indicators was good also in comparison with Italian and Portuguese intensive farms (14, 31) and Brazilian meat farms (18). Nevertheless, in one farm a high prevalence of animals with poor hair coat condition was found (24.7%): this result is in line with intensive farms, but it is higher than the prevalence found in extensive meat goats in Brazil (12.12%) (18). It is important to underline that in our protocol the assessment of hair coat condition was performed on individual animals during milking and not as a group-level indicator with goats free to move in the pen as proposed by the AWIN protocol (17). The assessment of individual animals might be more accurate than the assessment in a group of animals; hence, we think that this change in data collection could have affected the prevalence of the indicator. As to the prevalence of abscesses, on average the situation is in line with abscesses found in Brazilian meat farms (9.84%), but in at least one of our farms a serious welfare problem is present, with 35% of animals with abscesses. Improper disbudding was actually a problem only in one farm, which was the only one to perform this practice. The high prevalence of this indicator in this farm (more than 1/4 of the animals) deserves attention, because residual horns (scurs) on the head of adult goats can press against the head or eye, causing lesions and pain. Furthermore, scurs may be caught in fences and pen partitions, causing injuries and stress (17). As for hair coat condition, the assessment of improper disbudding was modified from group-level to individual-level indicator. Again, it is possible that the prevalence is affected by this change, making the assessment more accurate.

Potential welfare issues were highlighted regarding body condition and resting synchronization. The percentage of animals presenting a normal body condition was low (67.9%, but with a farm showing only 25% of animals with normal body condition) if compared to the results obtained in extensive double-purpose systems in Portugal [89.4%; (20)] and in intensive dairy systems in Portugal [77.7%; (31)] and in Italy [80.2%; (8)]. No direct comparison can be made with meat goats assessed by Leite Oliveira et al. (18), as the authors used a 5-point scale system, ranging from very thin to very fat. However, excluding the extremes (very thin = 14.5% and very fat = 2.1% of the animals), 83.4% of the animals in that study presented a normal body condition, in line with the other above-mentioned studies (8, 19, 20, 31). The low percentage of goats in normal body conditions in our farms was totally determined by the percentage of very thin animals. The presence of very thin animals is a problem also in Italian intensive farms (8) and in meat goats (18), but not in Portuguese intensive farms where the major problem is represented by very fat goats (31). The authors of the research performed in Portuguese extensive farms did not report the prevalence of too thin or too fat animals; hence, no further comparisons can be made (19, 20). The high prevalence of very thin goats in our farms could be explained by the fact that goats were in mid-lactation stage, when body reserves had not recovered from the losses occurred during the previous peak of lactation yet (32), aggravated by the absence of supplementary feed offered to goats in five farms during the grazing period. The risk for low energy intake is higher at pasture compared to indoor conditions as it is not always easy to fulfill the energy requirements of dairy animals only through natural forage resources (11). Additionally, animals at pasture move (horizontally and vertically) more than in indoor conditions and may be exposed to extreme weather conditions, thus spending more energy and requiring body fat mobilization (13).

The second indicator that poses some welfare concerns is synchrony at resting, which was very low in our study with only on average 14.3% of goats that lied down simultaneously, and even some farms where the animals never rested at the same time. When a sheltered area, such as a thick vegetation cover, was available, most of the goats (95.1%) used it for resting. According to Zobel et al. (24), hiding spaces (e.g., woods, caves), possibly in elevated areas, are important environmental features that allow goats to express their natural behavioral repertoire. The quality of sheltered areas may influence goats' resting behavior; probably those offered to the goats in our study were not sufficient to guarantee a high level of simultaneous resting. Furthermore, Negretti et al. (33) found that goats in an outdoor yard moved more, but rested less compared to housed goats. Hence, we could hypothesize that the low percentage of resting animals is due to a high exploratory behavior and need for movement in the outdoor environment before going back to the barns. The low observed number of resting goats might be due also to the presence of guardian dogs that elicited a strong fear reaction, and the presence of houseflies that bothered goats and prevented them from resting. Last, it is worth noting that the moment when goats were observed might not be optimal for resting observations. Findings from studies on feral goats would support the time we selected for performing the observations on resting synchronization. In fact, the diurnal activity of feral goats is characterized by feeding for >50% of daytime, mainly at dawn and dusk, and resting for >20% of daytime, mainly at midday, with a higher resting time from March to October, which decreases toward midwinter (34). Unfortunately, to our knowledge, no information is present in the literature regarding domestic goats at pasture. According to Stephan (35), animals can modify their activities during the day, to adapt to the surrounding environment. This might support the idea that goats at pasture graze until they are satiated, and may shift the time devoted to resting, performing it only in the barn after the evening milking. Furthermore, we may hypothesize that milking routine and other activities occurring in commercial farms, such as the distribution of supplementary feed, can affect the daytime activities of goats and, in this case, we may have missed the best timing for performing the observations on resting synchronization. In order to identify the best moment of the day to perform these observations, further research is needed to gather information about daytime activity budget and biorhythms of farmed goats. Therefore, in its present form, synchrony at resting is probably not suitable for inclusion in a welfare assessment protocol for goats, but certainly deserves further attention.

The AWIN welfare assessment protocol for goats (17) uses the Latency to first contact test to measure the quality of human-goat relationship. However, this test is not suitable for the assessment of the human-goat relationship when goats are at pasture. Hence, we used a different test, the FHAT, developed for the AWIN welfare assessment protocol for sheep, in order to check if it was suitable to evaluate the HAR quality in goats (16). However, since the validation of this test applied to goats is still pending, we used a simplified version, only considering the three possible reactions that goats could show, i.e., avoidance, contact and approach, and we did not record the distance expressed in meters when a flight response was elicited. The FHAT suggests that in most of the observed farms the relationship was positive, with goats voluntarily approaching the farmer in more than 60% of the farms. However, in more than 30% of the farms, the animals avoided any contact with the farmer. Caution should be used for the interpretation of these results since this test has not been validated for goats yet. Furthermore, comparisons with the results obtained in other extensively managed goats is difficult, due to differences in the procedures followed in other protocols for the evaluation of HAR. For example, in the Brazilian study on meat goats, an avoidance distance ranging from 57 to 239 cm was recorded, but it is unclear if only the avoidance distance was calculated, or if other possible reactions (approach, contact and avoidance) were assessed too (18).

QBA studies ground on contrasting expressive qualities where contexts are previously selected for their divergent characteristics (12). In our farm sample, farm characteristics are rather homogenous, and therefore the goats' expressive behavior on the farms showed a limited variation on the PCA plot, with only few exceptions. In most of the farms, the mood of the animals appeared to be positive, but the level of arousal cannot be clearly distinguished because Relaxed (low arousal) aligns with Lively and Aggressive (high arousal). An explanation for this can be the fact that Assessor A did not score any farm with Bored or Frustrated animals; hence, the evaluation is not complete and the QBA outcome was not very meaningful. QBA relies on the use of all the descriptors available in the list provided to the assessors: the absence of some descriptors influences the PCA plot, resulting in an uneven distribution of terms. In contrast with other studies [e.g., (36, 37)], QBA presented a low level of IOR, due to the insufficient agreement on PC1. QBA could be a feasible indicator to be used in semi-extensive systems (only 10 min of observations from one observation point), but our results suggest that the training provided during this trial was insufficient to obtain reliable results and that a more extensive training should be performed.

According to Kaufman and Rosenthal (38), IOR is frequently neglected in behavioral studies and, apart from studies on QBA (12), to our knowledge no studies have been conducted so far to investigate the IOR of welfare indicators collected on grazing goats. This issue is highlighted also by Richmond et al. (39) stating that the reliability of most of the physical and health indicators included in the AWIN welfare assessment protocol for sheep (16) was confirmed, but the majority of the behavioral indicators included in the same AWIN protocol (e.g., lying synchrony, human approach test) had not been tested for reliability before their inclusion in the protocol (39). Most of the indicators used in our research have been tested for reliability in intensive dairy goat farms in Portugal and Italy (40) and the results supported their inclusion in welfare assessment protocols for that specific context. However, these results cannot be automatically extended to semi-extensive conditions. Interestingly two of the indicators modified from the AWIN welfare assessment protocol (17), namely hair coat condition and improper disbudding that were originally collected as group-level indicator resulted in insufficient agreement among assessors when collected as individual-level indicator. This suggests the importance of training the assessors and testing IOR when some changes occur (e.g., context, data collection).

Most of the indicators collected on goats at pasture showed acceptable reliability; however, IOR was not sufficient for QBA (as already discussed) and synchrony at grazing. Investigating the reasons for this result, we identified some issues related to the background and training of the observers, and to feasibility constraints. Regarding background and training, assessor A had a sound experience with goats, whereas assessors B and C only had a limited experience. This may have affected the effectiveness of training that possibly did not bridge the gap among the assessors and in turn affected the results of the observations. Furthermore, the assessors reported several hurdles during the collection of the indicators that may have affected the IOR and reduced the feasibility of the protocol. The application of the whole protocol under semi-extensive conditions took more time than the application in intensive farming conditions. In fact, the average estimated time required in intensive conditions is 90 min/farm and 30–45 s/goat (17), whereas the application of the protocol in the present study required more than 4 h/farm and 3 min/goat for the individual assessment, and according to the assessors this was exhausting and time-consuming. When more than 15 lactating goats are present, according to the AWIN protocol the use of a sampling strategy for the individual-level assessment is recommended, where the sample size depends on the number of goats in the herd (14). However, in our study this strategy was not applied due to the small size of farms included in our research but, in the light of the results on the duration of the whole assessment, the recommendation to adopt a sampling strategy has to be kept in mind in the future in order to improve the feasibility of the assessment, especially in presence of large herds. This might allow reducing the time required for the application of the whole protocol, lasting possibly <2 h/farm and <5 min/animal. In fact, during a stakeholder consultation carried out within the AWIN project, farmers, veterinarians and technicians reported as acceptable for on-farm welfare evaluation a total time not exceeding 2 h and an individual assessment time of maximum 5 min per animal (8). The assessment of meat goat farms in Brazil ranged from 1 to 3 h, but the authors assumed that this time could increase with a greater number of animals (18). In this study a maximum of 50 goats were assessed in extensive systems. Furthermore, it has to be considered that the total time of application of the protocol may depend on the time needed to reach the grazing area that in some cases can be distant from the farm.

A further complication that affected the feasibility of our assessment, and in turn its reliability, is that observations performed when the animals were at pasture required the use of binoculars, in particular in unfenced pastures where animals could stray far away. If animals graze in areas with thick vegetation cover, observations can be difficult, as the vegetation reduces the visibility. As to visibility, the farms were visited on purpose only on good weather days, as we supposed that rain or fog could worsen the reliability of the results. Hence, we suggest checking the weather forecast before scheduling the farm visits.

In addition, the assessors reported difficulties to perform the individual assessment during milking in narrow and dark pens, especially because milking frequently occurred very early in the morning, under suboptimal lighting conditions. Hair coat condition, abscesses and udder asymmetry were considered the hardest indicators to be collected. A relatively low IOR was reached for improper disbudding in some cases (0.20), whereas a good IOR was obtained in other cases (0.91); the assessors did not report any specific constraints, probably because this indicator was only applicable on one farm. The assessors reported some difficulties to detect severely lame goats due to the different flooring on which the animals walked: in some cases, they walked on concrete floor, but in others, they reached the pasture on gravel roads with variable slopes. Different surfaces (e.g., hard or soft) and, in this case, also different slopes, may affect the reliability of the observation, as suggested by other authors (41). Although our original plan was to evaluate lameness on individual animals at the end of milking, while they were leaving the milking area, this turned out not to be feasible as, being milked in narrow pens, the animals did not have enough space to walk for a sufficient distance to be properly assessed. Therefore, we decided to evaluate it observing the group of animals while they were moving from the milking area to pastures. According to assessors B and C, the detection of severe lameness in large groups of goats moving together was not easy, but assessor A reported this as an optimal situation, as severely lame goats can easily be identified as they walk slower than the others. The absence of severely lame goats (and of overgrown claws) in our farms is in agreement with the results obtained in meat goats extensively raised in Brazil (18), suggesting a positive effect of pasture on the health of claws, as observed by de Morais (20). The beneficial effect of grazing for reducing lameness was observed also in cattle by several authors [e.g., (42, 43)]. The assessors also found difficulties in assessing the synchrony at grazing using the scan and instantaneous sampling method, due to the high number of animals that moved at the same time. The IOR among the three assessors was insufficient and further training seems necessary to make the collection of this indicator more reliable. Furthermore, the wide range of confidence intervals for this indicator (−0.44 < CI <0.93) suggested that the reliability is likely to be affected by the group size, the environment (e.g., presence of woods) and the distance from the animals. For synchrony at resting very good IOR was reached, probably due to the low number of animals that lied down simultaneously and because they did not move when resting.

Further studies are needed to test the validity of the FHAT in goats, but all the assessors reported that this test is easy to be conducted with goats at pasture and the agreement among assessors was perfect for the three possible reactions of goats to the farmer. However, it is probably advisable to register the goat reaction toward a familiar human when the farmer really gathers the flock, in order not to affect the routine and management of the farm, as maybe goats would react differently if handled out of the normal routine. In any case, the validity of this indicator in goats still has to be confirmed and requires further consideration.

All the indicators used in this attempt to adapt an already existing welfare assessment protocol for goats are animal-based measures. However, no practical animal-based indicator was found to cover the “absence of prolonged thirst” criterion. This lack is common to most of the evaluations conducted at pasture (11). However, Morales et al. (44) used skin elasticity and enophthalmia as signs of dehydration in cattle kept in silvopastoral systems. Indicators of dehydration (e.g., skin tent test, capillary refill time, thirst index) are available for some species [e.g., calves (45), horses (46), camels (47)], but not for goats. Hence, further research is needed to identify suitable indicators to assess this criterion. Although farmers may not perceive thirst as a welfare issue on grazing animals because they eat fresh grass, prolonged thirst may represent a serious welfare problem. This is probably the case also in our farms, as 4 out of 13 farms did not provide water points during the grazing period, and this may represent a serious welfare problem, particularly during hot summers.

In conclusion, this research showed that most of the indicators selected to assess the welfare of goats in semi-extensive conditions could be applicable, even if most of them were originally developed for intensive conditions. For most indicators, an acceptable level of reliability was reached; however, further research is required in order to identify a complete set of robust indicators in this specific context. Finally, feasibility constraints should be taken carefully into account as they can affect the reliability of the evaluation. For example, the assessor may decide to collect the individual indicators during either morning or evening milking, choosing the moment when the light is higher, as a scarce illumination can negatively affect the results. Furthermore, it may be advisable to collect FHAT according to the farm routine, for example, when the farmer gathers the flock before entering the barn for the evening milking.

Specific research should be conducted on daily activities and biorhythms of farmed goats to select the best moment of observation for evaluating the synchrony during feeding and, in particular, during resting. This research highlighted the lack of animal-based indicators to assess the effect of prolonged thirst in semi-extensive conditions; hence, specific research is needed to fill in this gap.

## Data Availability Statement

The raw data supporting the conclusions of this article will be made available by the authors, without undue reservation.

## Ethics Statement

Ethical review and approval was not required for the animal study because according to the National Italian Law (D.L. 26/2014), no specific ethical approval was required, as no pain, suffering, distress or prolonged damage equivalent to or greater than that caused by the insertion of a needle was applied. Written informed consent was obtained from the owners for the participation of their animals in this study. Written informed consent was obtained from the individual(s) for the publication of any potentially identifiable images or data included in this article.

## Author Contributions

MG collected data and organized the database. MB and MG performed the statistical analysis. MB wrote the first draft of the manuscript. MR and SM wrote sections of the manuscript. LB supervised the work. All the authors contributed to conception and design of the study, manuscript revision, read, and approved the submitted version.

## Funding

The authors received funds for open access publication fees by Open APC Project provided by University of Milan.

## Conflict of Interest

The authors declare that the research was conducted in the absence of any commercial or financial relationships that could be construed as a potential conflict of interest.

## Publisher's Note

All claims expressed in this article are solely those of the authors and do not necessarily represent those of their affiliated organizations, or those of the publisher, the editors and the reviewers. Any product that may be evaluated in this article, or claim that may be made by its manufacturer, is not guaranteed or endorsed by the publisher.
